# Guiding Principles for the Practice of Integrative Physical Therapy

**DOI:** 10.1093/ptj/pzad138

**Published:** 2023-10-10

**Authors:** Catherine Justice, Marlysa B Sullivan, Cheryl B Van Demark, Carol M Davis, Matt Erb

**Affiliations:** Integrative Health and Wellness Division of the Department of Medicine, Hennepin Healthcare Systems, Minneapolis, Minnesota, USA; Empower Veterans Program, Atlanta Veterans Administration Health Care System, Atlanta, Georgia, USA; Physical Rehabilitation Services, Dignity Health Yavapai Regional Medical Center, Prescott, Arizona, USA; Department of Physical Therapy, University of Miami Miller School of Medicine, Miami, Florida, USA; The Center for Mind-Body Medicine, Washington District of Columbia, USA

**Keywords:** Integrative Health, Integrative Medicine, Physical Therapy, Integrative Physical Therapy, Integrative Physiotherapy, Whole Person, Whole Person Health, Whole Person Care, Whole Person Physical Therapy, Trauma-Informed Care, Complementary Therapies, Mind Body Medicine

## Abstract

Integrative health is an emerging specialty inside multiple disciplines within the medical community, yet the practice of integrative physical therapy remains undefined. This perspective paper suggests a set of guiding principles to support the role of physical therapy in integrative health. These guiding principles, including therapeutic partnership, whole person health, living systems, movement as an integrative experience, and salutogenesis, are described and explored in-depth as they relate to all aspects of patient care and clinician experience. These guiding principles are articulated within the context of social determinants of health and the interrelated roles that environment, trauma, stress, and lifestyle all play within an integrative physical therapy plan of care. Examples of current integrative physical therapy practices that embody these principles are described. The 5 guiding principles are designed to elicit interprofessional inquiry into how integrative health models can be applied to the art and science of physical therapy practice. The expansion of integrative health into the field of physical therapy has the potential to improve individual and population health, as integrative physical therapy can be used to address prevention, health promotion, primary care, and wellness while acknowledging the complex, dynamic, and interconnected nature of the human condition.

**Impact:**

This perspective article presents 5 guiding principles to establish a framework to define and shape the growing application of an integrative health model to physical therapy practice. These integrative physical therapy guiding principles aim to improve the quality of whole-person, patient-centered care.

## Introduction

Integrative health is an evidence-based healing-oriented approach that takes into account the whole person, emphasizes lifestyle and the therapeutic relationship between practitioner and patient, and makes use of all appropriate therapies.^1^ The National Center for Complementary and Integrative Health further defines integrative health as bringing conventional health care approaches (medication, physical rehabilitation, psychotherapy, etc.) and complementary health approaches (acupuncture, yoga, probiotics, etc.) together with an emphasis on treating the whole person.^2^ The inclusion of physical rehabilitation by the National Center for Complementary and Integrative Health as a conventional approach begs the question of how would a physical therapist move into an integrative health paradigm.

**Figure 1:**
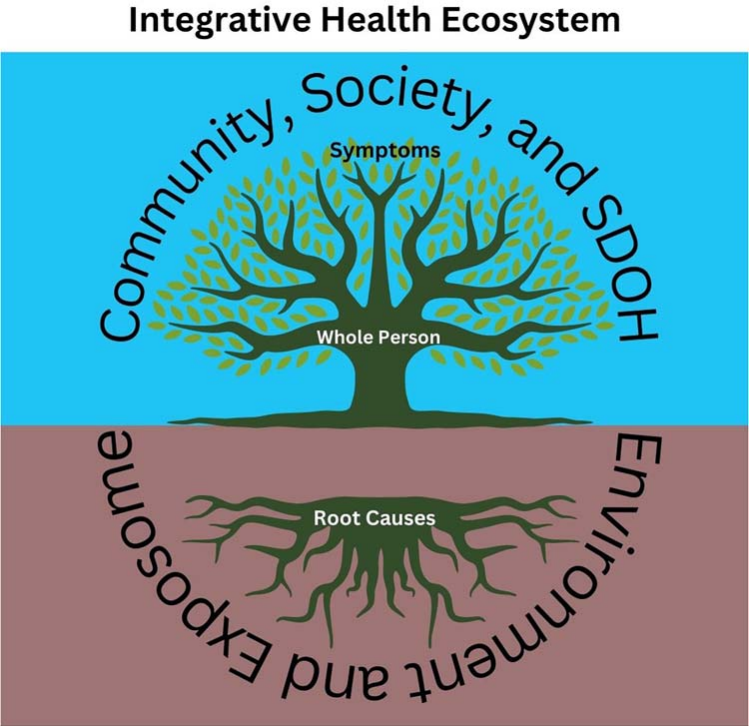
Integrative health ecosystem. SDOH = social determinants of health.

Integrative health is often conceptualized through a tree metaphor where a single symptom or condition is represented by a leaf or branch, the whole person and their full state of health and disease is the trunk, and the roots reflect deeper contributing factors. To extend the metaphor, the soil and air surrounding the tree represent the environment in which a person exists. This is further elucidated by the Center for Disease Control’s use of the term *exposome*, defined as a measure of all exposures from an individual’s diet, lifestyle, environment, etc. and how those exposures relate to their health and wellbeing.^3^ The metaphor can extend further to where the entire forest becomes representative of the larger society and culture, including social determinants of health (SDOH). When seeking integrative care, a patient can expect to explore multiple pathways of support throughout their whole tree and ecosystem (Fig. 1). Integrative health embraces a balanced approach to the 3-legged stool conceptualization of evidence-based medicine—giving each leg (patient preference, clinician expertise, and scientific evidence) appropriate weight.^4^

The mission of the American Physical Therapy Association is to advance the physical therapy profession toward the improvement of health for the whole of society with a vision of improving the individual human experience as part of the larger transformation of society through the optimization of movement.^5^ There is an emerging trend to integrate nutrition,^6^ prevention and wellness promotion,^7^ and population health,^8^ and the utilization of complementary and integrative health movement practices^9^^,^^10^ into physical therapy education and practice. Although the profession is embracing elements of integrative health care, the practice of Integrative Physical Therapy (IPT) requires a foundational shift in how care is administered.

**Figure 2:**
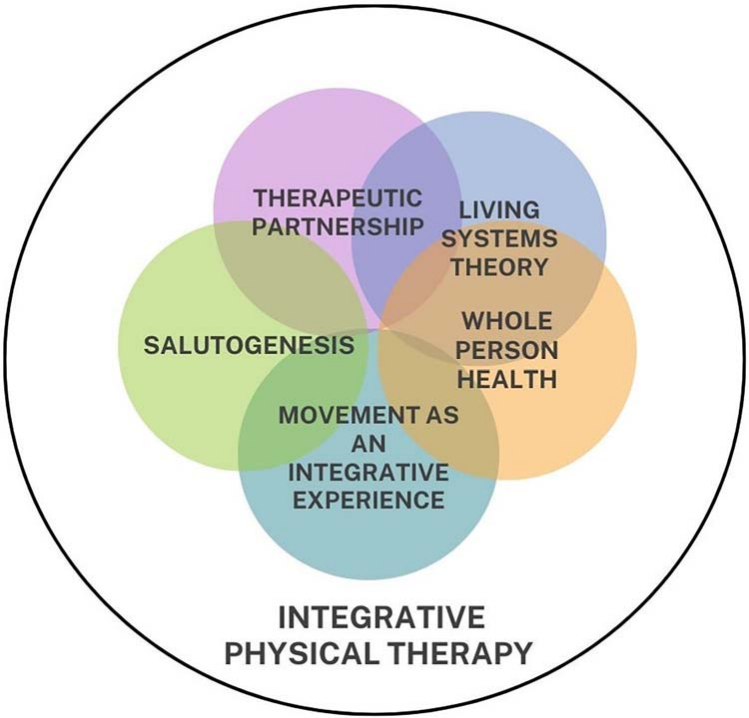
Integrative physical therapist guiding principles. Care model emphasizes interdependence of all aspects of each person’s experience including exposome and environment. Each Guiding Principle is held in awareness of clinical reasoning and care provision.

Integrative health approaches are increasingly sought out, with continued growth expected.^11^ The general principles of integrative health practices are defined as therapeutic partnership, recognition of mind, body, spirit, and community as factors that influence health, evidence-informed conventional and alternative healing practices, prioritizing less invasive interventions, openness to new paradigms of healing, health promotion and prevention, and the need for integrative health providers to personally embody these principles.^1^ Given a risk for the integrative health movement to inadvertently justify interventions that lack an evidence base, the practice of IPT calls for greater clarity via the creation of an established model that captures the unique perspectives of physical therapists. To that end, the authors propose a series of 5 specific guiding principles of integrative physical therapy practice as therapeutic alliance, whole person health (WPH), living systems theory, movement as an integrative experience, and salutogenesis (Fig. 2)**.** These principles are influenced by both modern, research-driven care models and ancient health systems.

## Guiding Principles for Integrative Physical Therapy

### Therapeutic Alliance

The clinician and patient relationship is central to the IPT tree. This relationship is conceived as a partnership (rather than a hierarchy) and encompasses values of person-centered, collaborative, and trauma-informed care (TIC). In this context, the integrative physical therapist serves as a caring guide for the person in the patient role. Treatment plans are created collaboratively with awareness of complex power dynamics, aligning the integrative physical therapist’s clinical judgment and skill within the patient’s expressed values, preferences, and intensions for seeking treatment.

Central to therapeutic alliance is the cultivation of the healthiest possible relational space.^12^ This is especially important for populations that have experienced systemic distrust in health care systems and historical disparities in services.^13^ In practice, therapeutic alliance relies on the clinician’s ability to engage in deep and embodied listening, garner trust, demonstrate cultural humility by seeing the relationship as a forum for mutual learning, and safely elicit a comprehensive patient narrative.^13^

There is evidence that history taking may be an effective pain reducer in and of itself.^14^ The integrative health model prioritizes time-specific strategies in support of this endeavor, including visit durations that are long enough to garner the trust for comprehensive history taking. When delivering physical therapist interventions within the larger context of humility, uncertainty, complexity of causality, and a purposefully attuned and caring relationship, the potential to meet implicit needs of the patient (such as to feel safe, to be seen, heard, validated, cared for, or empowered) increases.

This approach to the development of therapeutic alliance aims to also serve as support for provider wellbeing, where the provider no longer shoulders the weight of being the sole expert dictating the care of the patient. In a market study of integrative health physicians, 67% of doctors surveyed reported quality of life as much better or somewhat better since beginning to practice integrative medicine.^15^

### Trauma-Informed Care

A useful tool to leverage the first guiding principle of therapeutic alliance is the practice of TIC. TIC involves recognition of the effects of traumatic events and the broad spectrum of potential impacts that may arise out of past life experiences.^16^ A full set of considerations in regards to Adverse Childhood Experiences and TIC within rehabilitation care have been described.^17^ Examples of TIC within the context of IPT practice include requesting permission for any form of touch, maximizing patient empowerment by presenting options within each treatment session, and awareness of cultural humility, especially when working with persons from historically minoritized communities and/or when the physical therapist and the patient do not share the same cultural identity.^18^

### Whole Person Health

As the second guiding principle, WPH means considering the whole person—not just isolated organs or body systems—and honoring the complex factors that promote health or disease. WPH emphasizes individual and collective empowerment for wellbeing across the interconnected biological, behavioral, social, and environmental areas.^19^ IPT practice spotlights each of these factors in cocreating a WPH rehabilitation plan of care, with an understanding of the profound impact these factors can have on goals, prognosis, choice of healing modalities, etc. In this framework, the neuro-motor systems of the body are viewed as interdependent and intrinsically linked with all other body systems, alongside lifestyle and coping patterns, physical posture, environment and exposome, cognition, mental and emotional health, trauma and stress, culture, spirituality, and structural oppression.

A mutually agreed upon assessment of historical and lifestyle factors that may be influencing the person’s condition can be an integral part of IPT care. With skilled communication, an exploration of physical activity, nutrition, hydration, sleep, substance use, and social support carries potential for transformation of root factors potentially limiting wellbeing and optimal recovery. However, in alignment with the guiding principle of therapeutic alliance, it is imperative to approach the topic of lifestyle renewal with the utmost care, compassion, and contextual understanding. Many lifestyle choices can be rooted in adaptive coping arising out of unmet needs, historical experience, SDOH, and resource availability.

The broad set of SDOH are especially important to consider when working with historically marginalized groups such as persons who have been racialized, women, elderly, Indigenous populations, recent immigrants or refugees, and members of the lesbian, gay, bisexual, transgender, queer, intersex, and asexual communities. There is compelling evidence that experiences of discrimination are associated with worse health outcomes.^20^ Additional factors such as income, adverse childhood experiences, inadequate education, occupational and environmental hazard exposure, food access and security, zip code, neighborhood safety, and access to nature can all inform health trajectories toward or away from optimal health.^21^ Within a treatment visit, naming the influence of upstream and contextual determinants is a starting point to redistribute agency to the patient and put their struggles in a context that supports growth and empowerment. Specific strategies to further address SDOH in rehabilitation care have been delineated.^22^

#### Mind–Body Medicine

Consistent with WPH, integrative physical therapists are uniquely suited to support development of optimal body–mind–environment relationships. Within an integrative health model, the concept of mind–body medicine carries with it the intrinsic understanding that the mind and the body are not 2 separate interacting entities, but are rather intrinsically connected aspects of the whole of the human experience. In an IPT context, this translates to an understanding that you cannot treat the physical body without also affecting mental and emotional states (and vice versa). The development of body awareness and the use of imagery and biofeedback principles are core aspects of mind–body medicine and can be used to support empowerment and the development of a positive relationship with one’s physical body.^23^ Furthermore, IPT aims to engage an examination of sociological factors relevant to the physical therapist profession such as has been delineated by Nicholls,^24^ including contributing to structural change, applying critical inquiry towards personal, professional, and societal transformation, moving beyond WPH to whole community and even planetary health and well-being.

### Living Systems Theory

The third guiding principle, living systems theory explores the ways that living systems maintain themselves, interact, and adapt. Living systems are viewed as open (exchanging both energy and matter with the environment) and self-organizing (emergence of an overall order of a given system resulting from the collective interactions of individual components). In this way, living systems interact interdependently with their environment(s) and are dependent upon the interacting processes of multiple systems to survive and thrive.^25^ A dynamic living systems perspective demands tolerance of uncertainty as well as a skill set that optimizes strength-based inputs, carrying the potential to shift health trajectories toward greater well-being, regardless of the presence of injury, illness, or disease.^26^ Within the integrative health “tree” metaphor, integrative physical therapists are trained to look beyond the branch (symptom) level and dig into the roots (root causes), soil (environment and exposome), and forest (society and culture) and subsequently promote skills for enhanced resilience.

#### Biotensegrity

The biotensegrity model as an evolution of the reductive muscle and joint approach to understanding and treating musculoskeletal system dysfunctions is another concept that supports the IPT living system guiding principle.^27^ The bio-fascial-neuro-endocrine system is by nature integrative in that it encompasses, interweaves, and interpenetrates all organs, muscles, bones, and nerve fibers, giving the body functional structure, and enabling all body systems to operate in an integrated manner.^28^ The biotensegrity model takes into account the state of interdependence of all the body’s tissues in the transmission of force, the creation of movement and stillness, and the function of other systems throughout the body. Within an IPT perspective, understanding how the whole system responds to the demands placed on it becomes as equally important as the understanding of single joint and muscle strength and mobility tests and treatments.

#### Nervous System Regulation

As a second example of a living system in IPT practice, self-regulation provides a framework to explore how information is taken in, processed, and integrated into psychophysiological, somatoemotional, and biobehavioral responses. The autonomic nervous system is inseparably intertwined with the central nervous system and with every other system in the body, making the process of self-regulation of the stress response a holistic experience. As each organism is “hardwired” for the subconscious detection of danger (a process termed *neuroception* or threat appraisal), the central nervous system–autonomic nervous system –full body system functions on a continuum of mobilization and restoration.^29^ There are a number of evidence-informed theories (ie, polyvagal theory, neurovisceral integration theory, and the preparatory set) that underscore the relevance of approaching clinical care within a living systems perspective.^29–31^ Within living systems theory, we see that sources of stress and support can arise throughout the tree (individual body and mind), soil (environment and exposome), and ecosystem (society and culture)—at the levels of mental, emotional, physical, environmental, nutritional, social, and/or existential. Mitigation of stress through any one of these levels carries influence within the larger whole. Vital to the IPT model of care is the understanding of the complexities and uniqueness of how stress moves from healthy demand into toxic stress for each patient and collaboratively exploring stress management skills and resources. This lens, while potentially helpful for understanding all clinical presentations, is especially helpful in working to support patients with chronic disease, persistent pain, and states of multi-morbidity.

Through a living systems theory perspective, the integrative physical therapist can utilize an in-depth understanding of how such physiological responses impact symptomology, sensorimotor learning, behavior, and/or cognitive-emotional states. This aim includes considering how these self-regulatory states apply to movement interventions that elicit playfulness, joy, or personal connection (in contrast to conventional exercises). Both interoception (the ability to sense and regulate one’s internal state) and coregulation (the degree to which the state of one person’s nervous system regulation affects that of others) influence the experience of movement and are powerful tools that the integrative physical therapist can utilize within treatment sessions. Manual therapy, neuromuscular reeducation techniques, and therapeutic exercises can reflect an understanding of living systems theory when supporting safe body awareness and accurate interoception, cultivating regulated body–mind–behavioral states.^32^

When optimizing wellness through improved stress management is emphasized as part of an IPT care model, integrative physical therapists themselves also benefit. Embodying a positive lifestyle, practicing stress management, and utilizing integrative-minded movement practices can improve one’s own health and wellbeing and could serve as health care worker burnout prevention.^33^^,^^34^

### Movement as an Integrative Experience

As movement specialists, physical therapists aim to work with patients to develop nonableist and customized movement patterns and/or physical activity practices. With alignment to the patient’s history, motivations, and goals, this effort may include more frequent movement and/or prescribed doses of aerobic, resistance, flexibility, and neuromotor control activities.^35^ However, an IPT approach reframes physical activity into movement experiences informed by mindfulness research and social, environmental, and cultural factors.^36^^,^^37^ Integrative physical therapy supports examining energy management and balance in a holistic manner, including the relevance of rest and restorative processes.

It is vital in integrative physical therapy to explore each patient’s relationship to their body and how that translates into their relationship with movement. In this paradigm, movement may also be considered metaphorically. Am I moving backward or forward in life? Where is “movement” happening (or not happening) in my body? In my life as a whole? The integrative physical therapist approach also acknowledges and leverages how movement influences and is influenced by learning and cognition^38^; mental, emotional, and spiritual well-being^39^; stress and trauma biology^40^; social relationships^41^; and the attainment of various needs including instinctual and survival oriented impulses. The act of conscious breathing itself is considered a movement practice, promoting neurophysiological regulation and the relaxation response.^42^^,^^43^

As introduced in living systems theory, the science of interoception also underscores movement as an integrative experience, bringing insight into the relationships between the mind, brain, body, environment, and behavior.^44^ The development of self-referential processes around the integration of sensory signals with thoughts, beliefs, memories, intentions, posture, and movement can facilitate greater regulation and resilience.^45^^,^^46^ This interoceptive skill building can help the person to notice dysregulation that may stem from interacting physical, mental, social, environmental, etc. stressors, and refocus on what is aligned with meaning, purpose, and committed action in the service of one’s goals and values.

#### Mind and Body Movement Systems

Integrative physical therapy may include conventional forms of exercise alongside mind and body movement systems such as yoga, tai chi, qigong, dance, the Feldenkrais Method, the Alexander Technique, Laban Movement Analysis, etc. In response to the Flexner Report of 1910, the prevailing biomedical model of health categorized many ancient, indigenous, and culturally specific health practices as alternative medicine.^47^ In some cases, this categorization has created an unhelpful polarization and marginalization of many of the world’s established healing practices. The field of integrative health strives to offer an enhanced approach, wherein complementary and alternative medicine practices can be synergistically integrated with conventional care in a person-centered and evidence-based manner. The inclusion of ancient mind and body movement practices into physical therapy moves us away from these practices being marginalized as an “alternative” to conventional care, but rather as “integrated” into a model that includes both conventional and traditional healing practices.

### Salutogenesis

Integrative health models recognize that health and wellness are more than the absence of disease. *Salutogenesis* (health creation) is a concept that emphasizes facilitating a move toward greater well-being and flourishing, rather than solely moving away from illness, recognizing that even when aspects of an illness or injury remain, a person can continue to engage with life in a values-aligned way and experience well-being.^48^^,^^49^ Applying salutogenesis to the practice of integrative physical therapy means moving beyond restoring function or mobility toward a place where patients’ efforts in rehabilitation connect to personal concepts of flourishing within their current circumstance or situation.

#### Eudaimonia

As one means toward embracing a salutogenic framework, the Aristotelian concept of *eudaimonic well-being* refers to thriving within a well-lived life, a steadfast joy that does not fluctuate with circumstance.^50^ Within a salutogenic framework, eudaimonic wellbeing is defined as: meaning and purpose, self-defined virtues and ethics, social connection, autonomy, and personal expressiveness, and self-actualization and realization.^50^ Eudaimonic well-being has been explored and found to be connected to many positive health outcomes including: improved immune function, decreased allostatic load, decreased all-cause mortality independent of age, gender, physical inactivity, and the presence of disease.^51^^,^^52^ In chronic pain conditions, salutogenesis through eudaimonic well-being is related to lower levels of fatigue, disability, pain intensity, pain medication use, and to improved wellbeing, patient functioning, adjustment to chronic pain, depression symptoms, and life satisfaction.^53^^,^^54^

Within an IPT practice, salutogenesis means not just focusing on treating an injury or disorder, but encompassing interventions that encourage thriving. This could include mindfulness practices, healthy lifestyle, joyful movement, and/or interoceptive practices for improved mind and body connection.

### Integration of the Guiding Principles

It is essential to recognize that all 5 of the guiding principles (therapeutic partnership, WPH, living systems theory, movement as an integrative experience, and salutogenesis) are interdependent. For example, facilitating shifts in central nervous system–autonomic nervous system–full body regulation can impact how a person moves, their ability for social communication and relating, their pain experience, and more. Although systemic chronic inflammation is associated with a host of poor health outcomes and can be linked back to the interaction of SDOH with lifestyle (ie, poor nutrition, sedentary behavior, poorly managed stress, inadequate sleep, etc.),^55^ social experiences can also coregulate inflammation, ^56^ and social pain has shared physiological representations with physical pain.^57^ This highlights the overlap between WPH, living systems, and therapeutic partnership within IPT care. WPH itself incorporates a salutogenic approach, while also acknowledging the person as a living system. And TIC transcends the therapeutic partnership to include the recognition of trauma’s impact on the nervous system, the ability to self or coregulate, one’s relationship to movement, and contributions to WPH and salutogenesis, crossing through all 5 of the guiding principles.

#### Integrative Physical Therapy—From Theory to Practice

Some existing specialties within physical therapy lend themselves particularly well to an IPT approach. One example could be found in the field of pelvic health, where the sensitivity required to facilitate healing in the intimate areas of the pelvis and genitals aligns with elements of IPT care, such as trauma informed care, therapeutic partnership, and WPH. Physical therapists who work with people who experience chronic pain are another group that have a natural congruence with IPT. The biopsychosocial and biopsychosocial-spiritual approaches to chronic pain care have strong grounding in WPH and salutogenesis.^13^ Physical therapists working with people undergoing a cancer journey are also appropriate for this type of care. Navigating the physical, social, and emotional burden of cancer treatment has facilitated the field of integrative oncology, where understanding how to apply salutogenesis, healthier movement habits, and stress management strategies all contribute to long-term health, wellbeing, and survivorship.^58^ Evidence that the physical therapy profession can be inclusive of an integrative health approach can be seen in clinical practice guidelines based around preventative care, nutrition, yoga, etc.^59^^,^^60^

Although these clinical practice guidelines can be viewed as an emerging empirical evidence base for IPT care, the inclusion of preventative care, complementary and integrative health modalities, lifestyle education, etc. into an allopathic physical therapist paradigm is not equivalent to IPT care (Table). The shift to an integrative health paradigm requires that the 5 guiding principles are at the forefront of IPT patient care. The disintegrated existence of aspects of integrative health already present within the field of physical therapy indicates an even more pressing need for the profession to establish guiding principles. Physical therapists interested in this paradigm would benefit from a set of standards to guide professional development.

**Table:** Allopathic and Integrative Physical Therapy

Allopathic care	Focuses on treating symptoms. Physical therapist is the expert and determines the care plan in consultation with the patient. Reductive muscle and joint concept of movement. Healing injury or illness. Mental, emotional, and spiritual health are compartmentalized as separate from other systems. Treatment modalities center around conventional physical therapist practices. Complementary and integrative health and lifestyle education are possibly included as “add on” interventions.
Integrative care	Focuses on supporting the whole person. Model of mutual learning and collaborative care approach. Complex, multifaceted lens, and myofascial chains concept of movement Cultivates wellbeing and resilience and supports illness and injury prevention. Mental, emotional, and spiritual wellbeing are considered inseparable from physical wellbeing and thus are intrinsically part of physical therapist practice. Treatment approach may include conventional physical therapist practices alongside lifestyle and complementary and integrative health models.

An innovative example of an integrative physical therapy approach is currently offered at Hennepin Healthcare Systems in Minneapolis. Located in downtown Minneapolis, Hennepin Healthcare System includes a safety net hospital and outpatient clinics, providing care for low-income, the uninsured, and vulnerable populations. The integrative physical therapy specialty at Hennepin Healthcare System is described as a whole person approach to rehabilitation, focusing on maximizing the body’s ability to self-heal, nervous system regulation, and exploring mind, body, and spirit aspects of movement, helping patients uncover deeper compassion, joy, confidence, and purpose within their bodies.^61^ Sixty-minute treatment sessions allow for patient and IPT relationship building and wellness promotion through extensive subjective intakes that include inquiry around lifestyle, environment (safety and access to resources), social support, history of trauma, and SDOH. In this practice, the integrative physical therapists work in collaboration with the other integrative health practitioners in the organization, cofacilitating lifestyle-based group medical visits with integrative medical doctors, teaching trauma-sensitive yoga for the mother and baby mental health day hospital and cancer center, and practicing alongside acupuncturists and chiropractors. Although practices and modalities within the IPT specialty include conventional physical therapist practices, the therapists in this specialty carry additional training in mind–body science, yoga and yoga therapy, TIC, myofascial manual therapy, meditation, breathwork, and/or integrative medicine. In this case, each of the integrative health practices are more than an add-on modality, but rather inform the overall holistic paradigm of care within the specialty.

Another environment conducive to the IPT approach can be found the Veterans Health Administration Whole Health approach to care.^62^ This approach represents a systems-wide shift emphasizing health promotion, patient-driven care, and the integration of complementary and integrative health services along with conventional care. This includes a move toward asking “what matters to you, vs what is the matter with you.”^63^ There are 3 components to the Veterans Health Administration Whole Health System. The first of which is the Pathway, which includes empowering individuals to explore what matters to them through reflecting on one’s mission, aspiration, and purpose to set personalized goals for wellbeing. The second component includes well-being programs to support skills building of these practices—including complementary and integrative health approaches, other well-being approaches, and health coaching. Lastly, the Whole Health Clinical Care program integrates what matters to the veteran’s personalized goals and self-care, including healing environments and relationships, complementary and integrative health approaches, personal health planning, and health coaching. In the Veterans Health Administration, initiatives in physical therapy that incorporate these guiding principles in varying amounts include a biopsychosocial mentorship for physical therapists, codisciplinary pain care with physical therapists and behavioral health professionals, and the Tele-Pain-EVP Empower Veterans Program which centers on interdisciplinary care and the exploration of purpose and one’s identified values while supporting self-care skills building including self-regulation practices and WPH.^64^

### Challenges, Limitations, and Future Directions for IPT

Although a full discussion of the challenges and limitations to IPT care are out of the scope of this article, we recognize that deploying an IPT model of care demands both individual and structural effort. The dominance of our allopathic health care model creates myriad challenges when moving into IPT practice. From legal barriers for reimbursement for preventive or wellness-oriented care to limitations of diagnosis and referral frameworks that push physical therapists into the limited belief that they are treating a body part rather than the whole person, true integrative care requires transformation on individual and system levels. Similar to the efforts to advance physical therapy in mental health care, some might fear that facets of WPH are outside the scope of physical therapist practice. However, in the integrative health context, physical, mental, emotional, and spiritual wellbeing are all intrinsically connected; it is impossible to treat one without affecting the others. Clear delineation of scope of practice requires careful and ongoing discernment and clinical supervision and mentoring models are recommended.

IPT also necessitates treatment sessions with adequate time to build a therapeutic relationship between the physical therapist and the patient. Clinics that utilize shorter duration sessions will likely not be conducive to an IPT approach. Integrative physical therapists may also require additional time between patients to self-regulate in order to engage with each patient and client in a fully present state. This point raises an additional challenge to an IPT practice given that this work requires commitment to one’s own self-care, lifestyle, and mind and body practices.^1^

As there is an ever-growing public demand for a more holistic approach to health care, delineating the comprehensive mind–body skills that support IPT practice will establish physical therapy as an essential profession within the growing field of integrative health.^2^ The specifics on appropriate training and competencies for integrative physical therapy practice remain open. The American Medical Academy has set standards for physicians wishing to specialize in integrative health that include a 2-year fellowship program and an examination, among other requirements. The profession of physical therapy could benefit from examining the relevance of similar standards. Although a full exploration of standards for IPT practice are outside the scope of this article, determining the specifics for training and/or certification, including learning objectives, Commission on Accreditation in Physical Therapy Education standards, and continuing education certification curriculum could be a next step toward formalizing an IPT specialty. A more in-depth look at individual and structural barriers to IPT practice and how to overcome them have been delineated.^65^

Given the larger rapidly changing health care landscape, the physical therapy profession must address the question of whether integrative physical therapy should become a board-recognized clinical specialty versus seeing the principles that inform integrative physical therapy as a natural embodiment of the American Physical Therapy Association mission and vision of societal transformation. Either way, there is an urgent need for objective, evidence-based information and training on integrative physical therapy approaches and further elaboration and clinical models that integrate these principles.

### Conclusions

This paper proposes a foundation for integrative physical therapy practice with 5 interdependent guiding principles of therapeutic partnership, WPH, living systems, movement as an integrative experience, and salutogeneis. This perspective centers on the interdependence of all aspects of the person’s experience and supports the optimization of well-being with a focus on meaning, patient-identified values, and a purpose-filled life. Fully embracing an integrative health model as a physical therapist requires a deepened, mindful therapeutic presence, and a unique skill set to meet each proposed guideline within the dynamic complexities and challenges of modern health care. As we recognize that the health of all people is interdependent with ecological and planetary health, the need for an integrative approach to physical therapy that leverages these connections becomes even more pressing. This paper envisions a starting point of foundational guiding principles that constitute integrative physical therapist practice.

## Data Availability

Data sharing is not applicable to this article as no new data were created or analyzed in this Perspective.
